# Permeability of the landscape matrix between amphibian breeding sites

**DOI:** 10.1002/ece3.424

**Published:** 2012-11-08

**Authors:** Josh Buskirk

**Affiliations:** Institute of Evolutionary Biology & Environmental Studies, University of ZürichCH-8057, Zürich, Switzerland

**Keywords:** Amphibian, dispersal, landscape genetics, microsatellite markers, population structure, resistance

## Abstract

For organisms that reproduce in discrete habitat patches, land cover between patches (known as the matrix) is important for dispersal among breeding sites. Models of patchy populations often incorporate information on the permeability of the matrix to dispersal, sometimes based on expert opinion. I estimated the relative resistance to gene flow of land cover types and barriers using *F*_ST_ calculated from microsatellite markers in two amphibians, within an 800-km^2^ area in northern Switzerland. The species included a frog (*Rana temporaria*: 996 individuals, 48 populations, seven markers) and a newt (*Triturus alpestris*: 816 individuals, 41 populations, seven markers). Open fields and urban areas were more resistant to gene flow than forested land; roads and highways also reduced permeability. Results were similar for the two species. However, differences in resistance among matrix elements were relatively low: gene flow through urban areas was reduced by only 24–42% relative to forest; a divided highway reduced gene flow by 11–40% and was 7–8 times more resistant than a secondary road. These data offer an empirically based alternative to expert opinion for setting relative resistance values in landscape models.

## Introduction

For many organisms, the world consists of patches of habitat suitable for occupation separated by a matrix of uninhabitable space. This is a basic concept underpinning much of the early work in landscape ecology, biogeography, and metapopulation theory (Wiens 1995; Hanski 1999). Recent discoveries suggest that “the matrix matters” (Ricketts 2001), in the sense that variation in the composition of the unoccupied space between habitat patches, can influence populations within patches. This happens in several ways. Dispersal between pairs of patches may depend on the landscape elements, elevational gradients, and habitat types that fall between them (Cushman et al. 2006; Baguette and Van Dyck 2007). Alternatively, some species are not strictly confined to the habitat patch during their entire life cycle, and may use the matrix for foraging or hibernating. In such cases, it is not uncommon to observe that population density or occupation frequency of patches is related to the configuration of the matrix immediately surrounding the patches (Van Buskirk 2005; Ewers and Didham 2006; Angelone et al. 2011).

A major recent focus in landscape ecology is to estimate effects of the matrix on dispersal and patch occupation (Joly et al. 2001; Storfer et al. 2007, 2010; Minor and Urban 2008). The goal is to develop a better idea of when and how much the matrix matters. Which types of habitat or landscape elements act to obstruct dispersal, and by how much? What are the relative importances of land cover types? And to what extent do these differ among species? This study addresses these questions in a study of two amphibian species. The aim was to assign relative values to the permeability to gene flow of the basic types of landscape cover separating breeding sites, using data from the organisms themselves rather than external a priori information.

Amphibians that breed in water are well suited for this project because they depend on discrete wetlands for reproduction, but also utilize the surrounding habitat to varying degrees during the non-breeding season for foraging, hibernating, and dispersing. The species included in this study, *Rana temporaria* and *Triturus* (=*Mesotriton*) *alpestris*, are philopatric in the sense that most individuals return to breed in the same wetland in which they completed larval development, but there is nevertheless regular dispersal among distinct breeding sites (Perret et al. 2003; Palo et al. 2004; Safner et al. 2011). It has been shown in various amphibians that matrix habitat influences local population status (Carr and Fahrig 2001; Joly et al. 2001; Van Buskirk 2005) and the connectivity of populations (Spear et al. 2005; Murphy et al. 2010; Safner et al. 2011). However, we lack a quantitative picture of how much the matrix matters for gene flow among breeding sites: which are the landscape elements that most strongly impede movement, and how important are they relative to one another?

## Methods

The goal was to estimate the relationship between population connectedness and the composition of the landscape between populations. For the purposes of this study, a “population” was defined as the set of individuals breeding within a discrete wetland. The study had three stages: (1) the extent of dispersal among pairs of habitat patches was inferred indirectly from estimates of genetic divergence using neutral microsatellite markers; (2) the composition of the landscape between pairs of patches was measured from detailed maps of the study area; and (3) the relative contributions of types of landscape elements to population divergence were estimated using linear models. A strength of my approach is that information on resistance of landscape features to dispersal comes entirely from the organisms themselves. There was no initial step, as implemented in many other studies, of judging landscape permeability based on natural history information, behavioral observations, or expert opinion (e.g., Ray et al. 2002; Adriaensen et al. 2003; Cushman et al. 2006; Stevens et al. 2006; Compton et al. 2007; Storfer et al. 2007, 2010).

**Figure 1:**
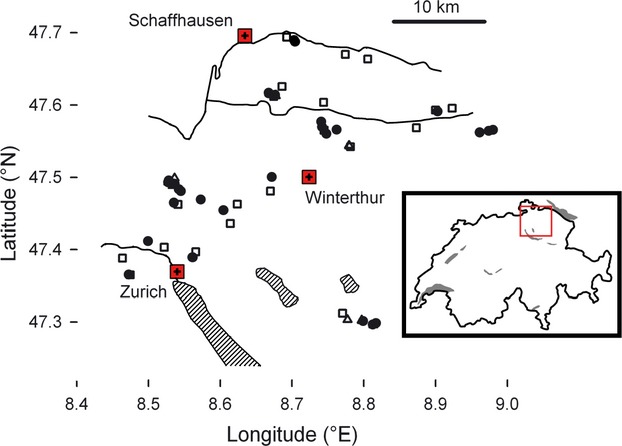
Map illustrating locations of the 61 ponds from which genetic samples were collected. The open squares contributed only *Rana temporaria*; open triangles only *Triturus alpestris*; filled circles both species. Rivers are indicated by lines, and lakes are hatched. The inset indicates the location of the study area within Switzerland. In some cases, ponds are so close together that their symbols cannot be distinguished; therefore, exact locations of all ponds are given in Table S1.

The habitat patches were wetlands supporting breeding aggregations of the common frog (*R. temporaria*) and alpine newt (*T. alpestris*), within an 800-km^2^ region of northern Switzerland (Fig. 1; Table S1). I studied only some of the many amphibian breeding localities within this region, chosen because of their accessibility for sampling or because I was able to secure permits for them. Unsampled populations do not severely bias estimates of migration rate among the sampled populations, according to Beerli's (2004) simulations, although Slatkin (2005) cautions that so-called “ghost populations” can be important under some circumstances.

### Neutral genetic samples

For *R. temporaria*, I collected one fertilized egg from each of at least 20 different clutches in each of 48 ponds in March 2000; 996 embryos were collected in total. Insofar as possible, half-sibs sired by the same male were avoided by sampling from clutches of different ages and in different parts of the pond. After tadpoles hatched and resorbed the yolk sac, they were stored in 96% ethyl alcohol until the DNA was extracted. The number of individuals genotyped per population averaged 20.7 (range 13–36; three populations had <17 samples). For *T. alpestris*, samples came from 816 larvae collected in 41 ponds by dip-netting or pipe-sampling during July 2000 (Van Buskirk 2009). Again, I avoided sampling relatives by distributing the dip-nets or pipe throws across large areas of the pond. The number of individuals per population averaged 19.9 (range 6–53). Tissue samples were stored in alcohol.

Amphibian larvae were genotyped at highly variable microsatellite loci, applying previously described protocols (Garner et al. 2003). There were eight loci for *R. temporaria* and seven loci for *T. alpestris*. One *R. temporaria* locus showed evidence for divergent selection, according to the test of Beaumont and Nichols (1996), and was therefore discarded from analyses. The markers and their statistical properties are described in Tables S2 and S3; Fig. S1 for *R. temporaria*, and in Garner et al. (2003), Table S4; Fig. S1 for *T. alpestris*. Both species exhibited some significant deviations from Hardy–Weinberg equilibrium, using exact probability tests (Raymond and Rousset 1995). Therefore, I estimated the frequency of null alleles following Brookfield (1996, eq. 2) and included the estimated frequencies as a single allele in subsequent analyses. Estimated null allele frequency averaged 0.084 for *R. temporaria* and 0.065 for *T. alpestris* (Tables S2 and S4).

### Landscape measures

Landscape features were measured along straight-line dispersal paths and within lens-shaped regions connecting all pairs of populations within 10 km of each other. I did not include population pairs >10 km apart for several reasons. First, evidence suggests that amphibians are philopatric or usually disperse a few hundred meters between the larval stage and first reproduction, only rarely covering kilometers (reviewed in Smith and Green 2005). In addition, there was significant isolation by distance in both species (Fig. S2). This implies that more distant population pairs, generally more than 5–10 km apart, were connected by dispersal only indirectly and over longer periods of time. Thus, there is a greater risk that mutation contributes to divergence between more distant populations. Finally, barriers and land cover become less relevant as distance increases and large numbers of different types of barriers accumulate (Murphy et al. 2010; Jaquiery et al. 2011).

For every allowed dispersal path, I measured the overall straight-line distance and the surface area of a lens-shaped region having a width 20% of the length and the ends anchored at the pair of ponds. For the lens regions, the density of distinct ponds and building structures was recorded. For the straight-line paths, I measured distances passing through three types of land cover: forest, open field, and urban (density of building structures ≥10 ha^−1^). I also counted the number of times the dispersal path traversed a secondary road, a divided highway, a river >5-m wide, an airport runway, or a rail line. These habitat and barrier types were chosen because distinctions among them have proven important in earlier work on amphibians (Angelone et al. 2011; Hether and Hoffman 2012). The landscape data were measured from digital versions of 1:25,000 topographic maps, updated between 1998 and 2003 (Bundesamt für Landestopographie, Wabern, Switzerland). Older maps confirm that, while land cover on the study area is not unchanged in recent decades, the basic configuration of ponds, forests, roads, and urban areas has remained consistent since the 1970s. This is especially true for forests, which are protected by Swiss federal law.

Analyses described below assume that animals follow (nearly) linear dispersal paths between breeding sites, a common assumption in landscape genetics (Storfer et al. 2010). Although linear dispersal cannot really occur, highly directed movement in the terrestrial habitat is often observed in radio-telemetry studies of amphibians (Matthews and Pope 1999; Freidenfelds et al. 2011) and linear dispersal is supported by statistical modeling (Spear et al. 2005; Goldberg and Waits 2010). In any case, comparison among indirect dispersal paths requires independent information about resistance of landscape elements (e.g., “least-cost modeling”; Adriaensen et al. 2003), and this would be incompatible with my aim of estimating resistance directly from data on gene flow.

### Statistical analyses

Interpopulation differentiation was estimated by *F*_ST_ using the allele identity method (Hardy and Vekemans 2002). *F*_ST_ is appropriate for this study because it indirectly reflects long-term migration rates between pairs of populations, under the assumption that divergence is more strongly influenced by drift than by selection and mutation (Slatkin 1991; Epperson 2005; Whitlock 2011). Although genetic effective population sizes (*N*_e_) are not known, annual counts of the number of clutches produced by female *R. temporaria* between 1999 and 2011 were fairly small (median 121, range 11–2315, *N* = 48 ponds). This suggests that drift may be more important for population divergence than mutation (Crow and Aoki 1984). Moreover, private alleles were infrequent (0.0012 in *R. temporaria* and 0.0046 in *T. alpestris*), and this too implies that divergence was not primarily due to new mutations. For both species, genetic divergence was far too low to directly estimate first-generation migrants (e.g., Beerli and Felsenstein 2001).

The number of individuals dispersing between each pair of populations per generation, *m*, was estimated according to Slatkin's (1993, eq. 6) formulation for two populations: *N*_e_*m* = (1/*F*_ST_ − 1)/4. Although the value of *N*_e_ is unknown, specific information on *N*_e_ would influence estimates of absolute dispersal, but not the relative impacts of landscape features on gene flow (see Discussion).

For each species, I constructed three types of linear model. The first predicted gene flow among population pairs based on the distance within the dispersal path covered by forest (*L*_F_), open field (*L*_O_), and urban (*L*_U_) land covers. The parameters of this model reflect the relative resistances to gene flow of the three kinds of land cover. The number of migrants between two populations, *i* and *j*, was expressed as:


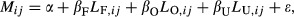
(1)

for all *i* < *j* (i.e., each population pair was included once). *M*_*ij*_ is the logarithm of *N*_e_*m*; α is the intercept, which estimates gene flow between immediately adjacent populations; the βs are coefficients representing the impact of a 1-km length of forest, open, or urban land; and ε is the variation in *M*_*ij*_ not explained by distances through the three land types.

The second model estimated the impact of discrete landscape elements – rivers, secondary roads, and highways – suspected to affect movement among populations:


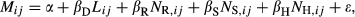
(2)

where the intercept α estimates gene flow between ponds that are immediately adjacent and have no landscape elements separating them; *L*_*ij*_ is the distance between populations *i* and *j* (km) (for all *i* < *j*); *N*_R_, *N*_S_, and *N*_H_ are the number of rivers, secondary roads, and divided highways falling between the populations; β_D_ is the change in gene flow per km; and the other βs are coefficients representing the impact of a single landscape element of the corresponding type. Railroad lines were combined with secondary roads and airport runways were combined with divided highways, because neither of these elements was sufficiently frequent to allow their contributions to be estimated separately. Convergence issues prevented me from including landscape elements and land cover within the same model, probably because multiple pairs of independent variables were highly correlated.

The third model asked whether gene flow was related to the densities of discrete building structures and wetlands falling within the lens-shaped region connecting pairs of populations:


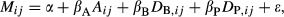
(3)

where α is the intercept, *A*_*ij*_ is the area of the lens-shaped region between populations *i* and *j* (ha); *D*_B_ and *D*_P_ are the densities of buildings and ponds falling within the lens-shaped area (per ha); β_A_ is the change in gene flow for each 1-ha increase in the area of the lens region; and the other βs are coefficients representing the impact of a change in the density of buildings and ponds.

These analyses were inspired by that in Ricketts (2001), modified here for use with data on neutral marker divergence. Parameters were estimated by maximum likelihood in SAS version 9.2 (SAS Institute 2009); confidence intervals and significance were evaluated from 9,999 permutations of the response variables in eqs 1–3.

## Results

**Figure 2:**
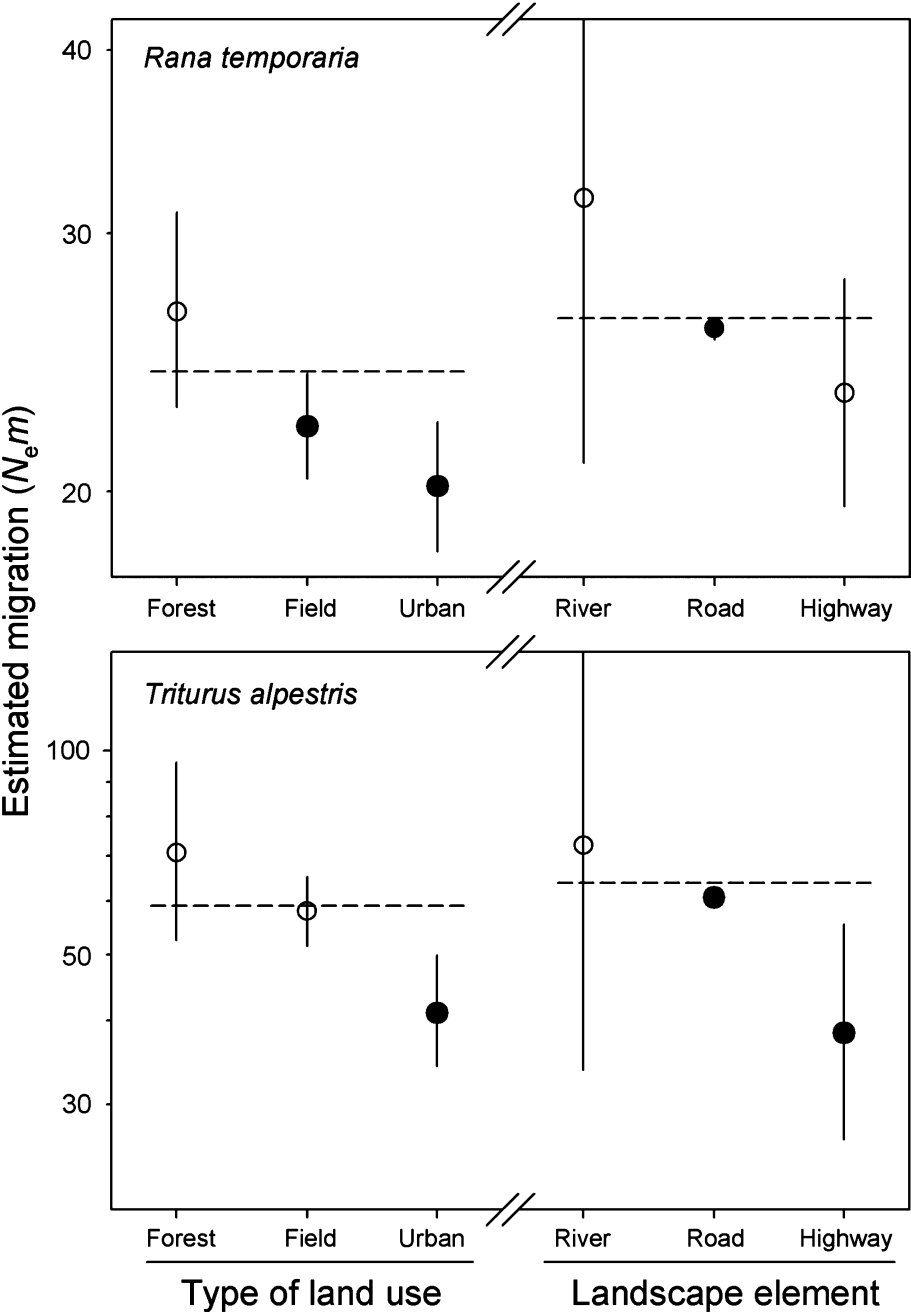
Impacts of land cover types (left side) and landscape elements (right side) on estimated gene flow between pairs of breeding populations for a frog (*Rana temporaria*) and a salamander (*Triturus alpestris*). The horizontal dashed line is the estimated number of migrants (*N*_e_*m*) between populations that are coincident (or immediately adjacent). The symbols and vertical lines illustrate the change in *N*_e_*m* (±95% CI) caused by the addition of 1-km land cover of the type indicated or the presence of one landscape element of the type indicated. Filled symbols emphasize impacts on gene flow that were significant in permutation tests.

*Triturus alpestris* showed higher rates of estimated gene flow than *R. temporaria* (Fig. 2). Analyses of land cover revealed significantly reduced migration rates across open fields and urban areas for *R. temporaria*, and through urban areas for *T. alpestris* (Table 1A). *N*_e_*m* among *R. temporaria* populations separated by urban land was reduced about 24% relative to that among populations separated by forest. That is, a standard distance through urban habitat permitted movement of 76% as many migrants as an equivalent distance of forested habitat. The corresponding figure for *T. alpestris* was a 42% reduction in gene flow caused by urban land (Fig. 2).

**Table 1:** Analyses of landscape impacts on gene flow among populations of two amphibian species

	*Rana temporaria*			*Triturus alpestris*		
		
Source	Estimate	P-value	95% CI	Estimate	P-value	95% CI
A. Type of land cover						
Intercept	3.185	0.0570		**4.079**	**0.0001**	
Forest	0.093	0.1185	−0.056, 0.250	0.180	0.1262	−0.118, 0.487
Field	−**0.086**	**0.0203**	−**0.169,** −**0.003**	−0.019	0.3885	−0.138, 0.099
Urban	−**0.181**	**0.0000**	−**0.283,** −**0.079**	−**0.365**	**0.0000**	−**0.547,** −**0.169**
B. Landscape element						
Intercept	**3.268**	**0.0215**		**4.156**	**0.0001**	
Distance (km)	−0.029	0.2028	−0.097, 0.038	**0.128**	**0.0127**	**0.018, 0.239**
Rivers	0.189	0.1781	−0.227, 0.583	0.128	0.3703	−0.637, 0.871
Secondary roads	−**0.016**	**0.0318**	−**0.034,** −**0.001**	−**0.050**	**0.0003**	−**0.082,** −**0.015**
Divided highways	−0.118	0.0955	−0.296, 0.061	−**0.511**	**0.0012**	−**0.873,** −**0.140**
C. Building and pond density						
Intercept	**3.346**	**0.0001**		**4.242**	**0.0001**	
Lens area (ha)	−**0.006**	**0.0173**	−**0.012, 0.001**	−0.001	0.4111	−0.008, 0.006
Building density	−**0.118**	**0.0075**	−**0.219,** −**0.015**	−**0.219**	**0.0001**	−**0.345, 0.060**
Pond density	−0.389	0.0703	−0.930, 0.472	0.008	0.6635	−0.070, 0.142

P-values and 95% confidence intervals come from 9999 permutations of the response variables (see eqs 1–3). Coefficients for lens area in part C are multiplied by 10. Samples sizes are 284 dispersal paths for *R. temporaria* and 183 paths for *T. alpestris*. Boldface highlights significant results.

Analyses of landscape elements revealed that roads and divided highways caused reduced gene flow, especially for *T. alpestris* (Table 1B). Reductions in *N*_e_*m* for each secondary road and divided highway were 1.6% and 11% in *R. temporaria*, and 4.9 and 40% in *T. alpestris* (Fig. 2). These figures agree with the observation that frogs cross roads somewhat better than salamanders (Hels and Buchwald 2001; Mazerolle et al. 2005). Movement among populations was not significantly influenced by the presence of an intervening river. The positive effect of distance in *T. alpestris* appeared only in models that included landscape elements; otherwise, gene flow declined significantly with distance in both species (*R. temporaria*: −0.068, *P* = 0.0099; *T. alpestris*: −0.054, *P* = 0.0393). This may be caused by the declining importance of landscape elements as distance increases (Murphy et al. 2010; Jaquiery et al. 2011).

Analysis of lens-shaped segments spanning pairs of populations confirmed that *N*_e_*m* declined with increasing distance – significantly so for *R. temporaria* – and with increasing density of building structures (Table 1C). Gene flow was entirely unaffected by the density of wetlands within the lens region. As in previous analyses, the relative magnitudes of coefficients confirmed that *T. alpestris* was more sensitive than *R. temporaria* to roads and urban land cover.

## Discussion

These results provide quantitative insight into resistance to gene flow among amphibian populations represented by different land use types and landscape elements. Although the level of gene flow was generally higher in *T. alpestris* than in *R. temporaria*, the two species experienced similar influences of the matrix between breeding sites. Forest cover was least resistant to movement and urban habitat most resistant. Roads obstructed gene flow in both species, with divided highways and airport runways about 7–8 times more resistant than secondary roads. These results are in broad agreement with information on habitat preferences of these species (Nöllert and Nöllert 1992), and with earlier work on how land cover and barrier structures modify exchange among populations of amphibians (Carr and Fahrig 2001; Spear et al. 2005; Murphy et al. 2010; Angelone et al. 2011).

The absolute levels of migration implied by my results are high, because effective population sizes in amphibians are usually quite small. Assuming that *N*_e_ is 100, which is on the high end of estimates from the literature (reviewed in Ficetola et al. 2010), the values of *N*_e_*m* depicted in Fig. 2 would be generated by long-term migration rates among adjacent (or coincident) populations in the range of 0.24 individuals per generation in *R. temporaria* and 0.59 individuals per generation in *T. alpestris*. A 1-km length of urban area would reduce these rates to 0.20 and 0.41 individuals per generation, respectively. Of course, these rates would increase if true values of *N*_e_ are smaller than 100.

The use of divergence at neutral genetic markers to indirectly reflect dispersal rate has important implications (Bossart and Prowell 1998; Whitlock 2011). On the positive side, genetic divergence reflects successful movement and reproduction (i.e., gene flow). Studies of marked animals cannot differentiate between individuals that disperse and breed, and those that merely wander or for some reason do not settle in the recipient habitat patch. Also, *F*_ST_ provides a measure of gene flow that can be applied to a large sample of populations and integrates over many generations (Whitlock 1992). Neither of these would be feasible in a mark-recapture study of individual movement (Koenig et al. 1996). On the other hand, *F*_ST_ scales with dispersal rate only under certain assumptions (Whitlock 2011). Two such assumptions, that markers are not under selection and that the mutation rate is smaller than the migration rate, are probably fulfilled in this study. Evidence against selection came from simulations showing that *F*_ST_ for individual microsatellite markers did not differ from that expected under neutrality. Evidence against high mutation rates came from the rarity of private alleles. Moreover, my focus on nearby population pairs helps ensure that migration exceeds mutation. Although there are limitations in the use of *F*_ST_ to infer gene flow, this study is at least in good company, because the great majority of analyses in landscape genetics have employed *F*_ST_ or its close relatives (Storfer et al. 2010).

Recent landscape models allow organisms to exhibit more realistic, non-linear dispersal paths between habitat patches. “Least-cost” models and their derivatives incorporate spatially explicit landscape information and produce detailed predictions about land use and movement paths (Adriaensen et al. 2003; Pinto and Keitt 2009). However, these approaches require independent knowledge about resistance of landscape features to animal movement, which comes from natural history information, behavioral observations of the organisms, or expert opinion (Spear et al. 2010). The same sources are used to supply a priori estimates of resistance for other forms of causal landscape modeling as well (Cushman et al. 2006; Stevens et al. 2006; Greenwald et al. 2009).

Estimates of landscape permeability based on data from the organisms themselves, rather than external observations, could be important for conservation planning and understanding landscape effects on population structure. Empirical estimates of relative resistance values across the three land cover types in my study are more similar to one another than are those proposed in the amphibian literature. For example, the resistance values used by Ray et al. (2002), Compton et al. (2007), and Greenwald et al. (2009) for fields and urban areas were 9–16 times higher than for forested land. Some studies of amphibians propose that urban land is entirely impermeable to dispersal (Stevens et al. 2006; Safner et al. 2011). In each of these cases, values were chosen based on tracking studies or knowledge of terrestrial habitat use, but this information need not reflect actual gene flow (Koenig et al. 1996; Bossart and Prowell 1998). My genetic estimates of the number of migrants per generation illustrate that urban areas and highways are indeed more resistant to gene flow than forested land, but that they are not nearly as resistant as previously assumed. More generally, the data suggest that permeability distinctions among land cover types, while statistically detectable in this and other studies, may be quantitatively less important than has been supposed (Ray et al. 2002; Stevens et al. 2006; Baguette and Van Dyck 2007; Safner et al. 2011). Of course, resistance estimates may differ among species with different habitat requirements (e.g., Stevens et al. 2006).

Conservation biologists find that estimates of landscape permeability are of practical use for parameterizing landscape models used to guide conservation strategy (Minor and Urban 2008; Leidner and Haddad 2011). Getting the permeability values right is important because the behavior of models is sensitive to the values chosen (Balkenhol et al. 2009; Rayfield et al. 2010). My approach is therefore valuable because it contributes to developing accurate parameters for use in basic and applied landscape and metapopulation models.

## Supporting Information

**Figure S1.** Results of Beaumont & Nichols–s test for selection for the *Rana temporaria* and *Triturus alpestris* microsatellite loci.

**Figure S2.** Isolation by distance among the 48 populations of *Rana temporaria* and 41 populations of *Triturus alpestris*.

**Table S1.** Locations of the populations of *Rana temporaria* and *Triturus alpestris* included in this study.

**Table S2.** Diagnostic statistics for microsatellite loci used to estimate *Rana temporaria* population divergence.

**Table S3.** Published sources for the *Rana temporaria* microsatellite loci, along with the number of non-null alleles occurring at each locus, *F*_ST_, and *F*_IS_.

**Table S4.** Diagnostic statistics for microsatellite loci used to estimate *Triturus alpestris* population divergence.
