# Supplementary material for: Permeability of the landscape matrix between amphibian breeding sites
Source: Ecol Evol. 2012 Nov 8;2(12):3160–7. doi: 10.1002/ece3.424 (PMC3539008; doi:10.1002/ece3.424)

Table S1. Locations and elevations of the populations of *Rana temporaria* and *Triturus alpestris* included in this study. Genetic samples were available for populations indicated with an "x."

Population	Latitude (°N)	Longitude (°E)	Elevation (m)	*Rana temporaria*	*Triturus alpestris*
allm	47.4833	8.5424	420	x	x
alls	47.4807	8.5451	422	x	x
amb1	47.3024	8.7970	540		x
amb3	47.3012	8.7990	538	x	x
amb4	47.3017	8.7974	539		x
anni	47.3882	8.4638	447	x	
awma	47.4621	8.5412	424	x	
birk	47.2964	8.8124	541	x	x
bode	47.5648	8.7446	498	x	
chaf	47.4034	8.5219	551	x	
chap	47.4118	8.4994	520	x	x
eige	47.4626	8.6238	507	x	
ente	47.6163	8.6675	400	x	x
eroa	47.6937	8.6926	397	x	
esch	47.6873	8.7042	404	x	x
etzw	47.6633	8.8055	430	x	
flgu	47.4883	8.5338	436		x
fucu	47.4926	8.5300	438		x
grab	47.4910	8.5363	429	x	x
grut	47.5617	8.9613	614	x	x
gurw	47.5599	8.7477	490	x	x
haup	47.4904	8.5350	434		x
hell	47.2959	8.8133	541		x
hiwi	47.2984	8.8169	559	x	x
home	47.4690	8.5727	488	x	x
hubs	47.3650	8.4745	628	x	
hubw	47.3653	8.4725	631	x	x
hund	47.3030	8.7774	509		x
insl	47.4956	8.5283	439	x	x
isol	47.4988	8.5363	426		x
jmai	47.4931	8.5277	440		x
jnw1	47.4930	8.5273	440	x	x
kebn	47.5419	8.7816	455	x	
kzwi	47.5639	8.9739	650	x	x
lang	47.5656	8.7621	472	x	x
mosl	47.5910	8.9027	460	x	x
mrbm	47.5431	8.7796	458		x
mrbn	47.5434	8.7793	458		x
mrbs	47.5426	8.7798	460		x
muet	47.4540	8.6045	458	x	x
oloo	47.5768	8.7406	465	x	x
opfi	47.5695	8.7417	515	x	x

oste	47.5682	8.8733	385	x	
pfac	47.6037	8.7439	470	x	
piro	47.6897	8.7030	419	x	x
rain	47.5956	8.9231	428	x	
raue	47.6126	8.6774	423		x
rauw	47.6131	8.6765	424	x	x
rode	47.6697	8.7744	529	x	
schl	47.4646	8.5353	434	x	x
seew	47.3122	8.7705	514	x	
stru	47.4808	8.6700	573	x	
sypf	47.4356	8.6141	470	x	
untr	47.3970	8.5658	591	x	
weck	47.5929	8.9000	455	x	
whof	47.6250	8.6862	401	x	
wolf	47.5654	8.9799	662	x	x
wtal	47.5003	8.6716	475	x	x
zaun	47.6120	8.6746	423	x	
zurl	47.3893	8.5617	645	x	x
zurs	47.3892	8.5613	645	x	x

Table S2. Diagnostic statistics for microsatellite loci used to estimate *Rana temporaria* population divergence. The table includes two parts. Part 1 shows population-level statistics for the eight loci, one of which was discarded from later analyses. For each locus, values are reported for the number of tadpoles scored (N), the number of alleles present not including null alleles (A), observed and expected heterozygosity (Ho and He) calculated without accounting for null alleles, and estimated null allele frequency (NAF; eqn. 2 in Brookfield 1996, *Molecular Ecology* 5:453-455). I estimated null allele frequencies because deviation from Hardy-Weinberg equilibrium was in some cases significant when checked with exact probability tests (Guo & Thompson 1992, *Biometrics* 48:361-372; implemented in GENEPOP 4.0.10). For all subsequent analyses, I adjusted allele frequencies at the population level, introducing one new allele under the assumption that all allelic dropout arose from a single null. Part 2 reports population means for A, Ho, and He after correcting for null alleles.

Part 1

Population	N	A	Ho	He	NAF
Locus RtCa22					
allm	18	2	0.167	0.157	-0.009
alls	18	2	0.000	0.108	0.097
amb3	20	2	0.050	0.050	0
anni	19	1	0.000	0.000	0
awma	23	1	0.000	0.000	0
birk	19	1	0.000	0.000	0
bode	20	3	0.100	0.188	0.074
chaf	18	2	0.056	0.056	0
chap	19	1	0.000	0.000	0
eige	17	2	0.059	0.059	0
ente	22	1	0.000	0.000	0
eroa	10	1	0.000	0.000	0
esch	23	2	0.000	0.085	0.078
etzw	20	1	0.000	0.000	0
grab	21	2	0.000	0.093	0.085
grut	21	4	0.095	0.403	0.220
gurw	25	2	0.120	0.115	-0.004
hiwi	19	1	0.000	0.000	0
home	22	2	0.091	0.089	-0.002
hubs	1	1	0.000	0.000	0
hubw	5	1	0.000	0.000	0
insl	19	2	0.158	0.149	-0.008
jnw1	19	2	0.053	0.053	0
kebn	18	1	0.000	0.000	0
kzwi	19	1	0.000	0.000	0
lang	22	1	0.000	0.000	0
mosl	22	2	0.045	0.045	0
muet	21	2	0.048	0.048	0
oloo	24	4	0.125	0.122	-0.003

opfi	24	1	0.000	0.000	0
oste	19	1	0.000	0.000	0
pfac	23	4	0.174	0.167	-0.006
piro	19	2	0.053	0.053	0
rain	22	3	0.091	0.090	-0.001
rauw	21	2	0.048	0.048	0
rode	14	1	0.000	0.000	0
schl	24	1	0.000	0.000	0
seew	21	1	0.000	0.000	0
stru	25	1	0.000	0.000	0
sypf	17	2	0.118	0.114	-0.004
untr	21	3	0.143	0.138	-0.004
weck	12	1	0.000	0.000	0
whof	30	1	0.000	0.000	0
wolf	16	3	0.125	0.232	0.087
wtal	21	1	0.000	0.000	0
zaun	18	1	0.000	0.000	0
zurl	19	3	0.105	0.104	-0.001
zurs	19	3	0.158	0.152	-0.005
average	19.1		0.045	0.062	0.014
Locus RtCa2-22					
allm	15	6	0.733	0.662	-0.043
alls	12	6	0.667	0.768	0.057
amb3	19	8	0.526	0.733	0.119
anni	13	6	0.769	0.766	-0.002
awma	19	7	0.632	0.688	0.033
birk	16	8	0.688	0.800	0.062
bode	15	8	0.467	0.669	0.121
chaf	10	8	0.600	0.789	0.106
chap	11	5	0.455	0.766	0.176
pfac	14	8	0.643	0.751	0.062
eige	5	5	0.400	0.711	0.182
ente	14	5	0.500	0.585	0.054
eroa	10	3	0.400	0.442	0.029
esch	19	5	0.526	0.485	-0.028
etzw	16	7	0.688	0.661	-0.016
grab	18	8	0.500	0.805	0.169
grut	16	7	0.563	0.607	0.027
gurw	17	7	0.765	0.709	-0.033
hiwi	16	7	0.563	0.716	0.089
home	9	4	0.444	0.647	0.123
hubs	10	6	0.900	0.774	-0.071
hubw	19	8	0.421	0.777	0.200
insl	17	5	0.588	0.663	0.045
jnw1	16	4	0.625	0.613	-0.007
kebn	11	7	0.818	0.788	-0.017
kzwi	16	6	0.438	0.647	0.127
lang	20	7	0.850	0.783	-0.038
mosl	19	8	0.737	0.751	0.008
muet	18	5	0.389	0.551	0.104
oloo	20	7	0.700	0.710	0.006
opfi	14	8	0.929	0.767	-0.092
oste	18	8	0.722	0.786	0.036
piro	9	5	1.000	0.673	-0.195
rain	17	4	0.588	0.599	0.007
rauw	14	5	0.357	0.563	0.132

rode	9	6	0.667	0.693	0.015
schl	14	6	0.643	0.603	-0.025
seew	18	7	0.611	0.622	0.007
stru	17	5	0.471	0.469	-0.001
sypf	10	5	0.700	0.595	-0.066
untr	18	6	0.833	0.811	-0.012
weck	17	9	0.765	0.786	0.012
whof	32	5	0.594	0.622	0.017
wolf	17	5	0.706	0.640	-0.040
wtal	15	7	0.733	0.667	-0.040
zaun	14	4	0.714	0.521	-0.127
zurl	13	6	0.385	0.732	0.200
zurs	9	6	0.333	0.765	0.245
average	14.8		0.626	0.686	0.034

Locus RtCa9 (discarded from analyses; see figure below)

allm	18	7	0.389	0.708	0.187
alls	18	10	0.833	0.852	0.010
amb3	21	9	0.762	0.789	0.015
anni	19	7	0.684	0.745	0.035
awma	23	9	0.565	0.643	0.047
birk	19	10	0.947	0.829	-0.065
bode	20	5	0.600	0.581	-0.012
chaf	19	8	0.789	0.765	-0.014
chap	21	11	0.762	0.815	0.029
pfac	22	6	0.591	0.541	-0.032
eige	15	7	0.667	0.763	0.054
ente	9	4	0.222	0.399	0.127
eroa	7	5	0.714	0.659	-0.033
esch	19	6	0.526	0.744	0.125
etzw	18	8	0.333	0.830	0.272
grab	16	6	0.375	0.661	0.172
grut	19	8	0.474	0.787	0.175
gurw	22	7	0.545	0.550	0.003
hiwi	18	7	0.611	0.757	0.083
home	20	11	0.650	0.842	0.104
hubs	18	7	0.389	0.663	0.165
hubw	21	7	0.381	0.724	0.199
insl	17	5	0.176	0.701	0.309
jnw1	18	4	0.611	0.494	-0.078
kebn	13	6	0.615	0.606	-0.006
kzwi	15	9	0.600	0.846	0.133
lang	20	8	0.450	0.612	0.100
mosl	23	6	0.130	0.702	0.336
muet	16	10	0.875	0.768	-0.061
oloo	22	9	0.636	0.689	0.031
opfi	24	6	0.375	0.429	0.038
oste	13	9	0.769	0.874	0.056
piro	20	8	0.750	0.741	-0.005
rain	18	10	0.778	0.871	0.050
rauw	21	9	0.762	0.735	-0.016
rode	13	6	0.615	0.751	0.078
schl	23	9	0.609	0.646	0.022
seew	21	9	0.810	0.660	-0.090
stru	19	11	0.737	0.787	0.028
sypf	16	6	0.750	0.776	0.015
untr	21	10	0.714	0.828	0.062

weck	14	7	0.071	0.817	0.411
whof	33	9	0.697	0.758	0.035
wolf	19	9	0.579	0.799	0.122
wtal	21	9	0.667	0.576	-0.058
zaun	17	8	0.588	0.743	0.089
zurl	13	3	0.308	0.532	0.146
zurs	17	10	0.588	0.651	0.038
average	18.2		0.572	0.706	0.078
Locus RtCa30					
allm	18	11	0.667	0.854	0.101
alls	17	8	0.118	0.807	0.381
amb3	19	8	0.421	0.745	0.186
anni	18	7	0.333	0.644	0.189
awma	18	8	0.167	0.875	0.378
birk	15	9	0.467	0.802	0.186
bode	14	8	0.357	0.852	0.267
chaf	17	6	0.412	0.681	0.160
chap	18	8	0.500	0.784	0.159
pfac	22	12	0.636	0.819	0.101
eige	13	7	0.538	0.745	0.119
ente	17	6	0.353	0.804	0.250
eroa	8	5	0.250	0.667	0.250
esch	20	8	0.500	0.826	0.179
etzw	16	10	0.438	0.800	0.201
grab	16	11	0.375	0.849	0.256
grut	15	10	0.467	0.887	0.223
gurw	24	9	0.375	0.838	0.252
hiwi	19	6	0.421	0.632	0.129
home	21	9	0.619	0.848	0.124
hubs	20	9	0.350	0.710	0.211
hubw	20	12	0.450	0.865	0.223
insl	17	8	0.412	0.750	0.193
jnw1	15	6	0.267	0.506	0.159
kebn	6	3	0.500	0.439	-0.042
kzwi	16	9	0.625	0.841	0.117
lang	21	9	0.619	0.800	0.101
mosl	17	9	0.235	0.824	0.323
muet	19	10	0.526	0.767	0.136
oloo	23	8	0.435	0.611	0.109
opfi	23	8	0.304	0.703	0.234
oste	16	8	0.500	0.825	0.178
piro	16	7	0.375	0.750	0.214
rain	18	7	0.611	0.762	0.086
rauw	21	8	0.286	0.806	0.288
rode	10	7	0.400	0.842	0.240
schl	22	9	0.273	0.832	0.305
seew	17	9	0.235	0.786	0.309
stru	22	9	0.364	0.809	0.246
sypf	14	9	0.214	0.862	0.348
untr	19	7	0.316	0.673	0.213
weck	19	7	0.421	0.829	0.223
whof	32	11	0.531	0.854	0.174
wolf	15	8	0.333	0.834	0.273
wtal	18	8	0.333	0.816	0.266
zaun	17	8	0.294	0.802	0.282
zurl	17	7	0.412	0.734	0.186

zurs	17	9	0.529	0.701	0.101
average	17.5		0.406	0.768	0.203

Locus RtCa2-28

allm	18	9	0.667	0.817	0.083
alls	16	9	0.563	0.817	0.140
amb3	21	6	0.381	0.748	0.210
anni	17	6	0.471	0.697	0.133
awma	23	9	0.478	0.697	0.129
birk	18	7	0.556	0.784	0.128
bode	20	7	0.550	0.797	0.137
chaf	16	9	0.563	0.847	0.154
chap	14	6	0.357	0.804	0.248
pfac	18	6	0.278	0.798	0.289
eige	17	9	0.588	0.865	0.149
ente	19	8	0.579	0.791	0.118
eroa	10	8	0.900	0.853	-0.025
esch	18	8	0.500	0.813	0.173
etzw	14	7	0.429	0.812	0.211
grab	21	7	0.667	0.749	0.047
grut	16	6	0.250	0.659	0.247
gurw	25	9	0.560	0.755	0.111
hiwi	20	10	0.500	0.878	0.201
home	21	7	0.429	0.799	0.206
hubs	21	6	0.524	0.751	0.130
hubw	24	8	0.667	0.772	0.059
insl	14	7	0.643	0.783	0.079
jnw1	18	8	0.667	0.606	-0.038
kebn	17	8	0.353	0.838	0.264
kzwi	17	6	0.176	0.713	0.313
lang	20	8	0.600	0.812	0.117
mosl	14	7	0.286	0.772	0.274
muet	19	6	0.684	0.667	-0.010
oloo	24	10	0.625	0.784	0.089
opfi	21	9	0.762	0.830	0.037
oste	18	7	0.333	0.849	0.279
piro	19	7	0.789	0.832	0.023
rain	18	3	0.333	0.656	0.195
rauw	9	5	0.889	0.771	-0.067
rode	8	6	0.500	0.783	0.159
schl	22	8	0.545	0.815	0.149
seew	10	6	0.400	0.726	0.189
stru	22	8	0.682	0.683	0.001
sypf	15	7	0.467	0.630	0.100
untr	21	7	0.429	0.702	0.160
weck	13	5	0.231	0.778	0.308
whof	13	5	0.308	0.815	0.279
wolf	18	5	0.111	0.673	0.336
wtal	20	7	0.750	0.810	0.033
zaun	14	6	0.500	0.683	0.109
zurl	18	7	0.500	0.735	0.135
zurs	16	8	0.500	0.716	0.126
average	17.4		0.516	0.764	0.140

Locus Rtempµ8

allm	18	9	0.778	0.681	-0.058
alls	15	5	0.200	0.409	0.148

amb3	21	9	0.571	0.706	0.079
anni	19	9	0.368	0.684	0.188
awma	21	10	0.381	0.490	0.073
birk	18	10	0.556	0.683	0.075
bode	15	5	0.133	0.361	0.168
chaf	18	9	0.556	0.675	0.071
chap	20	7	0.450	0.465	0.010
pfac	22	8	0.636	0.555	-0.052
eige	16	7	0.438	0.520	0.054
ente	21	10	0.571	0.578	0.004
eroa	10	5	0.500	0.511	0.007
esch	21	11	0.857	0.736	-0.070
etzw	18	10	0.667	0.711	0.026
grab	21	8	0.524	0.487	-0.025
grut	21	7	0.571	0.630	0.036
gurw	23	5	0.435	0.410	-0.018
hiwi	19	4	0.158	0.154	-0.003
home	21	12	0.667	0.692	0.015
hubs	21	11	0.762	0.746	-0.009
hubw	24	10	0.458	0.469	0.007
insl	21	8	0.524	0.685	0.096
jnw1	19	6	0.632	0.579	-0.034
kebn	18	7	0.333	0.433	0.070
kzwi	19	9	0.632	0.597	-0.022
lang	21	7	0.429	0.382	-0.034
mosl	22	12	0.727	0.770	0.024
muet	20	8	0.600	0.533	-0.044
oloo	20	10	0.650	0.605	-0.028
opfi	20	7	0.200	0.436	0.164
oste	21	13	0.762	0.791	0.016
piro	17	6	0.294	0.371	0.056
rain	21	11	0.667	0.626	-0.025
rauw	21	9	0.667	0.678	0.007
rode	8	6	0.500	0.617	0.072
schl	24	10	0.750	0.672	-0.047
seew	17	7	0.471	0.570	0.063
stru	21	6	0.238	0.381	0.104
sypf	14	9	0.714	0.728	0.008
untr	21	10	0.810	0.744	-0.038
weck	20	9	0.550	0.771	0.125
whof	33	10	0.515	0.740	0.129
wolf	18	7	0.556	0.537	-0.012
wtal	21	9	0.476	0.490	0.009
zaun	18	9	0.611	0.651	0.024
zurl	19	10	0.737	0.687	-0.030
zurs	19	11	0.684	0.727	0.025
average	19.3		0.542	0.583	0.027
Locus Rtempµ7					
allm	15	7	0.467	0.777	0.174
alls	7	4	0.571	0.648	0.047
amb3	14	8	0.643	0.828	0.101
anni	17	8	0.353	0.759	0.231
awma	22	7	0.591	0.816	0.124
birk	19	7	0.421	0.733	0.180
bode	10	5	0.500	0.789	0.162
chaf	16	4	0.438	0.448	0.007

chap	13	7	0.538	0.806	0.148
pfac	19	8	0.474	0.842	0.200
eige	11	5	0.364	0.563	0.127
ente	15	6	0.600	0.671	0.042
eroa	6	5	0.667	0.833	0.091
esch	21	10	0.810	0.800	-0.006
etzw	16	8	0.500	0.728	0.132
grab	16	7	0.563	0.720	0.091
grut	16	10	0.875	0.841	-0.018
gurw	22	10	0.909	0.756	-0.087
hiwi	18	9	0.667	0.697	0.018
home	12	7	0.500	0.761	0.148
hubs	20	10	0.850	0.728	-0.071
hubw	25	8	0.800	0.826	0.014
insl	21	10	0.762	0.864	0.055
jnw1	19	11	0.684	0.841	0.085
kebn	17	10	0.941	0.875	-0.035
kzwi	20	10	0.650	0.795	0.081
lang	18	10	0.778	0.762	-0.009
mosl	19	9	0.737	0.802	0.036
muet	18	7	0.556	0.670	0.068
oloo	22	9	0.591	0.768	0.100
opfi	23	12	0.783	0.851	0.037
oste	21	7	0.524	0.776	0.142
piro	19	7	0.895	0.828	-0.037
rain	20	9	0.550	0.735	0.107
rauw	19	8	0.789	0.775	-0.008
rode	8	6	0.625	0.767	0.080
schl	23	9	0.478	0.594	0.073
seew	16	4	0.188	0.506	0.211
stru	12	7	0.500	0.645	0.088
sypf	14	5	0.643	0.751	0.062
untr	19	9	0.842	0.797	-0.025
weck	18	9	0.778	0.800	0.012
whof	31	10	0.581	0.776	0.110
wolf	18	8	0.500	0.756	0.146
wtal	19	6	0.632	0.720	0.051
zaun	18	10	0.778	0.789	0.006
zurl	14	8	0.643	0.783	0.079
zurs	11	8	0.364	0.779	0.233
average	17.1		0.614	0.751	0.079
Locus RtCa25					
allm	16	10	0.688	0.837	0.081
alls	12	6	0.583	0.783	0.112
amb3	3	3	1.000	0.733	-0.154
anni	13	8	0.462	0.754	0.166
awma	22	16	0.864	0.922	0.030
birk	16	8	0.500	0.819	0.175
bode	20	12	0.450	0.883	0.230
chaf	15	12	0.733	0.869	0.073
chap	18	13	0.611	0.892	0.149
pfac	16	12	0.750	0.913	0.085
eige	12	8	0.500	0.786	0.160
ente	17	9	0.529	0.859	0.178
eroa	9	7	0.778	0.837	0.032
esch	21	14	0.857	0.892	0.018

etzw	18	10	0.833	0.875	0.022
grab	19	14	0.947	0.925	-0.011
grut	18	12	0.944	0.854	-0.049
gurw	23	13	0.696	0.905	0.110
hiwi	14	15	0.786	0.931	0.075
home	9	8	0.556	0.902	0.182
hubs	13	13	0.538	0.948	0.210
hubw	23	14	0.652	0.831	0.098
insl	18	8	0.667	0.763	0.054
jnw1	19	10	0.632	0.826	0.106
kebn	17	17	0.941	0.950	0.005
kzwi	21	14	0.810	0.877	0.036
lang	22	12	0.773	0.845	0.039
mosl	20	15	0.750	0.918	0.088
muet	12	8	0.500	0.764	0.150
oloo	22	13	0.818	0.888	0.037
opfi	18	13	0.500	0.916	0.217
oste	19	15	0.842	0.879	0.020
piro	16	11	0.500	0.859	0.193
rain	22	14	0.909	0.910	0.001
rauw	20	9	0.750	0.859	0.059
rode	10	11	0.500	0.895	0.208
schl	22	18	0.682	0.927	0.127
seew	21	13	0.667	0.893	0.119
stru	7	6	0.286	0.879	0.316
sypf	15	12	0.733	0.846	0.061
untr	19	11	0.789	0.903	0.060
weck	8	9	0.500	0.917	0.218
whof	24	12	0.708	0.850	0.077
wolf	12	10	0.667	0.866	0.107
wtal	19	13	0.684	0.885	0.107
zaun	14	8	0.714	0.775	0.034
zurl	16	11	0.688	0.847	0.086
zurs	17	11	0.765	0.852	0.047
average	16.5		0.689	0.867	0.095

Part 2

Population	A	Ho	He
	Population averages		
allm	8.1	0.63	0.70
alls	6.6	0.44	0.69
amb3	6.9	0.57	0.67
anni	7.0	0.45	0.66
awma	9.0	0.51	0.67
birk	7.9	0.52	0.70
bode	7.7	0.44	0.70
chaf	7.7	0.51	0.65
chap	7.3	0.47	0.67

pfac	8.9	0.58	0.71
eige	6.9	0.56	0.66
ente	7.0	0.49	0.64
eroa	4.9	0.49	0.61
esch	8.6	0.51	0.68
etzw	8.0	0.61	0.68
grab	8.7	0.54	0.70
grut	8.4	0.56	0.73
gurw	8.1	0.60	0.65
hiwi	7.9	0.59	0.60
home	7.6	0.48	0.71
hubs	8.3	0.54	0.67
hubw	9.1	0.69	0.67
insl	7.4	0.53	0.70
jnw1	7.0	0.56	0.60
kebn	7.9	0.55	0.66
kzwi	8.4	0.58	0.67
lang	8.0	0.53	0.63
mosl	9.3	0.59	0.71
muet	7.0	0.55	0.61
oloo	9.1	0.51	0.66
opfi	8.7	0.59	0.67
oste	8.9	0.55	0.72
piro	6.7	0.58	0.65
rain	7.6	0.57	0.65
rauw	7.0	0.55	0.67
rode	6.7	0.51	0.70
schl	9.1	0.52	0.65
seew	7.3	0.43	0.63
stru	6.4	0.42	0.57
sypf	7.4	0.54	0.67
untr	7.9	0.62	0.70
weck	7.4	0.55	0.71
whof	8.3	0.51	0.68
wolf	7.1	0.52	0.69
wtal	7.6	0.54	0.64
zaun	6.7	0.54	0.62
zurl	8.0	0.54	0.69
zurs	8.6	0.55	0.71

Table S3. The number of non-null alleles (A) occurring at each locus for *Rana temporaria*, along with measures of population divergence (F_{ST}; allele identity method of Cockerham, 1973, *Genetics* 74:679-700) and inbreeding (F_{IS}), calculated in GENEPOP 4.0.10 under the assumption that a single null allele exists.

Locus	A	F_{ST}	F_{IS}	Reference**
RtCa22	6	0.041	0.217	Primmer & Merilä 2002
RtCa2-22	12	0.035	0.097	Teacher et al. 2009
RtCa9*	24	0.083	0.164	Garner & Tomio 2001
RtCa30	20	0.022	0.407	Teacher et al. 2009
Rt2Ca2-28	16	0.029	0.279	Teacher et al. 2009
Rtempµ8	28	0.023	0.077	Rowe & Beebee 2001
Rtempµ7	20	0.021	0.142	Rowe & Beebee 2001
RtCa25	36	0.032	0.139	Lesbarreres et al. 2005
Average		0.036	0.193	
Average without RtCa9		0.027	0.201	

* Discarded from analyses because of evidence for selection (see Fig. S1).

** References:

Garner, T. W. J., and G. Tomio. 2001. Microsatellites for use in studies of the Italian Agile Frog, *Rana latastei*. Conservation Genetics 2:77-80.

Lesbarreres, D., C. R. Primmer, A. Laurila, and J. Merilä. 2005. Environmental and population dependency of genetic variability-fitness correlations in *Rana temporaria*. Molecular Ecology 14:311-323.

Primmer, C. R., and J. Merilä. 2002. A low rate of cross-species microsatellite amplification success in ranid frogs. Conservation Genetics 3:445-449.

Rowe, G., and T. J. C. Beebee. 2001. Polymerase chain reaction primers for microsatellite loci in the common frog *Rana temporaria*. Molecular Ecology Notes 1:6-7.

Teacher, A. G. F., T. W. J. Garner, and R. A. Nichols. 2009. Population genetic patterns suggest a behavioral change in wild common frogs (*Rana temporaria*) following disease outbreaks (Ranavirus). Molecular Ecology 18:3163-3172.

Table S4. Diagnostic statistics for microsatellite loci used to estimate *Triturus alpestris* population divergence. The table includes three parts. Part 1 shows population-level statistics for the seven loci. For each locus, values are reported for the number of larvae scored (N), the number of alleles present not including null alleles (A), observed and expected heterozygosity calculated without accounting for null alleles (Ho and He), and estimated null allele frequency (NAF; eqn. 2 in Brookfield 1996, *Molecular Ecology* 5:453-455). I estimated null allele frequencies because deviation from Hardy-Weinberg equilibrium was in some cases significant when checked with exact probability tests (Guo & Thompson 1992, *Biometrics* 48:361-372; implemented in GENEPOP 4.0.10). For all subsequent analyses, I adjusted allele frequencies at the population level, introducing one new allele under the assumption that all allelic dropout arose from a single null.

Part 2 reports population means for A, Ho, and He.

Part 3 reports the number of non-null alleles (A) occurring at each locus, along with measures of population divergence (F_{ST}; allele identity method of Cockerham [1973, *Genetics* 74:679-700]) and inbreeding (F_{IS}), calculated in GENEPOP under the assumption that a single null allele exists.

Part 1

Population	N	A	Ho	He	NAF
Locus Ta1Ca1					
allm	10	4	0.600	0.553	0.000
alls	15	5	0.733	0.687	0.000
amb1	20	7	0.500	0.617	0.072
amb3	28	6	0.357	0.604	0.154
amb4	19	5	0.474	0.587	0.071
birk	9	3	0.556	0.542	0.000
chap	23	3	0.273	0.406	0.095
ente	20	4	0.316	0.397	0.058
esch	19	2	0.316	0.273	0.000
flgu	19	4	0.526	0.539	0.008

fucu	20	4	0.300	0.347	0.035
grab	42	4	0.634	0.552	0.000
grut	6	2	0.500	0.530	0.020
gurw	18	3	0.389	0.398	0.006
haup	21	5	0.500	0.586	0.054
hell	20	6	0.550	0.577	0.017
hiwi	14	5	0.714	0.624	0.000
home	23	6	0.609	0.614	0.003
hubw	20	3	0.550	0.573	0.015
hund	22	5	0.500	0.543	0.028
insl	21	5	0.600	0.544	0.000
isol	20	6	0.500	0.487	0.000
jmai	17	5	0.412	0.497	0.057
jnw1	20	2	0.500	0.431	0.000
kzwi	18	5	0.500	0.605	0.065
lang	20	7	0.474	0.549	0.048
mosl	27	3	0.481	0.408	0.000
mrbm	20	2	0.400	0.385	0.000
mrbn	21	5	0.571	0.566	0.000
mrbs	22	5	0.500	0.518	0.012
muet	20	4	0.600	0.637	0.023
oloo	20	5	0.400	0.491	0.061
opfi	21	5	0.476	0.519	0.028
piro	20	2	0.350	0.358	0.006
raue	7	2	0.143	0.143	0.000
rauw	53	5	0.321	0.371	0.036
schl	19	3	0.471	0.469	0.000
wolf	7	2	0.429	0.363	0.000
wtal	18	4	0.611	0.529	0.000
zurl	16	4	0.625	0.504	0.000
zurs	21	4	0.333	0.417	0.059
average	19.9		0.478	0.496	0.025

Locus Ta2Caga3

allm	10	11	0.700	0.842	0.077
alls	15	17	0.800	0.949	0.076
amb1	20	13	0.684	0.724	0.023
amb3	28	17	0.778	0.739	0.000
amb4	19	11	0.632	0.775	0.081
birk	9	10	0.889	0.902	0.007
chap	23	13	0.696	0.833	0.075
ente	20	18	0.632	0.747	0.066
esch	19	17	0.789	0.906	0.061
flgu	19	20	0.789	0.858	0.037
fucu	20	17	0.800	0.877	0.041
grab	42	27	0.854	0.933	0.041
grut	6	6	0.667	0.758	0.052
gurw	18	12	0.611	0.746	0.077
haup	21	17	0.800	0.892	0.049
hell	20	15	0.850	0.885	0.019
hiwi	14	9	0.643	0.632	0.000
home	23	18	0.739	0.850	0.060
hubw	20	13	0.800	0.882	0.044
hund	22	13	0.864	0.850	0.000
insl	21	15	0.905	0.801	0.000
isol	20	13	0.700	0.863	0.087
jmai	17	11	0.824	0.775	0.000

jnw1	20	18	0.800	0.903	0.054
kzwi	18	14	0.889	0.902	0.007
lang	20	15	0.700	0.865	0.088
mosl	27	18	0.852	0.809	0.000
mrbm	20	13	0.750	0.758	0.005
mrbn	21	14	0.714	0.784	0.039
mrbs	22	16	0.682	0.678	0.000
muet	20	16	0.650	0.881	0.123
oloo	20	18	0.850	0.844	0.000
opfi	21	14	0.667	0.844	0.096
piro	20	12	0.600	0.831	0.126
raue	7	9	0.571	0.868	0.159
rauw	53	27	0.736	0.862	0.068
schl	19	15	0.842	0.832	0.000
wolf	7	11	0.857	0.956	0.051
wtal	18	14	0.778	0.911	0.070
zurl	16	13	0.563	0.821	0.142
zurs	21	16	0.810	0.868	0.031
average	19.9		0.750	0.835	0.050
Locus Ta3Caga2					
allm	10	2	0.500	0.521	0.014
alls	15	2	0.533	0.497	0.000
amb1	20	5	0.450	0.671	0.132
amb3	28	4	0.481	0.544	0.041
amb4	19	3	0.684	0.531	0.000
birk	9	3	0.667	0.582	0.000
chap	23	4	0.261	0.305	0.034
ente	20	2	0.778	0.508	0.000
esch	19	2	0.579	0.508	0.000
flgu	19	2	0.526	0.444	0.000
fucu	20	3	0.550	0.499	0.000
grab	42	2	0.463	0.409	0.000
grut	6	2	0.667	0.485	0.000
gurw	18	3	0.200	0.434	0.163
haup	21	3	0.450	0.481	0.021
hell	20	2	0.500	0.508	0.005
hiwi	14	6	0.357	0.775	0.235
home	23	4	0.522	0.538	0.010
hubw	20	3	0.250	0.296	0.035
hund	22	3	0.455	0.507	0.035
insl	21	2	0.429	0.345	0.000
isol	20	2	0.500	0.492	0.000
jmai	17	4	0.625	0.591	0.000
jnw1	20	3	0.350	0.409	0.042
kzwi	18	2	0.444	0.457	0.009
lang	20	3	0.412	0.508	0.064
mosl	27	3	0.333	0.498	0.110
mrbm	20	3	0.421	0.444	0.016
mrbn	21	4	0.429	0.501	0.048
mrbs	22	4	0.500	0.532	0.021
muet	20	3	0.300	0.492	0.129
oloo	20	2	0.500	0.467	0.000
opfi	21	2	0.476	0.372	0.000
piro	20	2	0.579	0.491	0.000
raue	7	2	0.714	0.495	0.000
rauw	53	2	0.538	0.493	0.000

schl	19	4	0.375	0.556	0.116
wolf	7	2	0.571	0.527	0.000
wtal	18	3	0.389	0.500	0.074
zurl	16	2	0.533	0.460	0.000
zurs	21	2	0.714	0.494	0.000
average	19.9		0.5	0.500	0.033
Locus Ta4Caga4					
allm	10	3	0.700	0.695	0.000
alls	15	7	0.467	0.662	0.117
amb1	20	4	0.375	0.685	0.184
amb3	28	5	0.542	0.636	0.057
amb4	19	5	0.474	0.673	0.119
birk	9	3	0.667	0.680	0.008
chap	23	5	0.364	0.455	0.063
ente	20	5	0.600	0.678	0.046
esch	19	9	0.579	0.772	0.109
flgu	19	5	0.789	0.713	0.000
fucu	20	6	0.250	0.644	0.240
grab	42	7	0.700	0.706	0.004
grut	6	6	0.500	0.833	0.182
gurw	18	6	0.250	0.678	0.255
haup	21	6	0.450	0.713	0.154
hell	20	4	0.300	0.642	0.208
hiwi	14	6	0.500	0.732	0.134
home	23	7	0.522	0.746	0.128
hubw	20	7	0.700	0.787	0.049
hund	22	5	0.722	0.617	0.000
insl	21	6	0.550	0.712	0.095
isol	20	4	0.737	0.656	0.000
jmai	17	8	0.600	0.768	0.095
jnw1	20	6	0.529	0.679	0.089
kzwi	18	6	0.444	0.732	0.166
lang	20	6	0.588	0.734	0.084
mosl	27	4	0.556	0.645	0.054
mrbm	20	4	0.350	0.665	0.189
mrbn	21	8	0.737	0.768	0.018
mrbs	22	7	0.636	0.737	0.058
muet	20	3	0.700	0.682	0.000
oloo	20	6	0.474	0.708	0.137
opfi	21	7	0.286	0.647	0.219
piro	20	5	0.722	0.708	0.000
raue	7	4	0.429	0.692	0.155
rauw	53	9	0.735	0.727	0.000
schl	19	5	0.313	0.563	0.160
wolf	7	6	0.429	0.703	0.161
wtal	18	6	0.500	0.694	0.115
zurl	16	7	0.533	0.740	0.119
zurs	21	6	0.400	0.672	0.163
average	19.9		0.53	0.690	0.101
Locus Ta3Caga1					
allm	10	14	1.000	0.947	0.000
alls	15	16	0.933	0.947	0.007
amb1	20	22	0.550	0.964	0.211
amb3	28	22	0.846	0.953	0.055
amb4	19	16	0.706	0.913	0.108

birk	9	12	0.889	0.941	0.027
chap	23	18	0.895	0.939	0.023
ente	20	18	0.632	0.949	0.163
esch	19	18	0.684	0.939	0.132
flgu	19	18	1.000	0.952	0.000
fucu	20	18	0.750	0.940	0.098
grab	42	24	0.625	0.926	0.156
grut	6	9	0.667	0.939	0.140
gurw	18	14	0.611	0.932	0.166
haup	21	22	0.800	0.960	0.082
hell	20	22	0.850	0.959	0.056
hiwi	14	14	0.667	0.935	0.139
home	23	19	0.571	0.951	0.195
hubw	20	16	0.800	0.913	0.059
hund	22	19	0.818	0.948	0.067
insl	21	26	0.810	0.957	0.075
isol	20	18	0.947	0.932	0.000
jmai	17	18	0.625	0.956	0.169
jnw1	20	18	0.650	0.945	0.152
kzwi	18	18	0.611	0.952	0.175
lang	20	15	0.667	0.933	0.138
mosl	27	26	0.815	0.963	0.075
mrbm	20	14	0.850	0.914	0.033
mrbn	21	18	0.810	0.945	0.069
mrbs	22	18	0.952	0.940	0.000
muet	20	19	0.900	0.955	0.028
oloo	20	21	0.650	0.933	0.146
opfi	21	25	0.850	0.969	0.060
piro	20	17	0.750	0.937	0.097
raue	7	8	0.333	0.879	0.291
rauw	53	29	0.941	0.955	0.007
schl	19	21	0.684	0.959	0.140
wolf	7	8	0.714	0.901	0.098
wtal	18	19	0.722	0.929	0.107
zurl	16	18	0.800	0.954	0.079
zurs	21	21	0.714	0.913	0.104
average	19.9		0.760	0.940	0.096
Locus Ta6Ca1					
allm	10	4	0.300	0.611	0.193
alls	15	5	0.333	0.405	0.051
amb1	20	4	0.263	0.383	0.087
amb3	28	4	0.217	0.527	0.203
amb4	19	6	0.235	0.643	0.248
birk	9	4	0.222	0.464	0.165
chap	23	4	0.308	0.514	0.136
ente	20	5	0.176	0.629	0.278
esch	19	5	0.167	0.563	0.253
flgu	19	4	0.133	0.577	0.282
fucu	20	5	0.111	0.656	0.329
grab	42	6	0.293	0.427	0.094
grut	6	3	0.200	0.556	0.229
gurw	18	5	0.077	0.452	0.258
haup	21	4	0.188	0.280	0.072
hell	20	4	0.167	0.475	0.209
hiwi	14	3	0.182	0.416	0.165
home	23	4	0.333	0.489	0.105

hubw	20	4	0.235	0.608	0.232
hund	22	4	0.300	0.528	0.149
insl	21	4	0.286	0.400	0.081
isol	20	4	0.333	0.665	0.199
jmai	17	5	0.313	0.675	0.216
jnw1	20	5	0.188	0.470	0.192
kzwi	18	5	0.188	0.563	0.240
lang	20	5	0.059	0.519	0.303
mosl	27	5	0.391	0.612	0.137
mrbm	20	5	0.350	0.663	0.188
mrbn	21	5	0.429	0.647	0.132
mrbs	22	4	0.333	0.511	0.118
muet	20	4	0.158	0.494	0.225
oloo	20	5	0.471	0.640	0.103
opfi	21	4	0.067	0.545	0.309
piro	20	5	0.263	0.615	0.218
raue	7	4	0.400	0.600	0.125
rauw	53	5	0.458	0.666	0.125
schl	19	3	0.389	0.475	0.058
wolf	7	3	0.429	0.560	0.084
wtal	18	4	0.111	0.508	0.263
zurl	16	4	0.154	0.557	0.259
zurs	21	5	0.278	0.592	0.197
average	19.9		0.260	0.540	0.183
Locus Ta3Ca8					
allm	10	4	0.700	0.721	0.012
alls	15	8	0.571	0.722	0.088
amb1	20	4	0.385	0.689	0.180
amb3	28	7	0.680	0.733	0.031
amb4	19	4	0.444	0.652	0.126
birk	9	5	0.500	0.725	0.130
chap	23	7	0.667	0.745	0.045
ente	20	6	0.579	0.696	0.069
esch	19	7	0.737	0.734	0.000
flgu	19	4	0.722	0.687	0.000
fucu	20	6	0.316	0.627	0.191
grab	42	7	0.600	0.716	0.068
grut	6	5	0.500	0.652	0.092
gurw	18	5	0.231	0.625	0.242
haup	21	7	0.611	0.744	0.076
hell	20	5	0.632	0.673	0.025
hiwi	14	5	0.556	0.712	0.091
home	23	6	0.750	0.736	0.000
hubw	20	5	0.700	0.714	0.008
hund	22	8	0.524	0.675	0.090
insl	21	4	0.571	0.587	0.010
isol	20	4	0.700	0.676	0.000
jmai	17	9	0.667	0.798	0.073
jnw1	20	4	0.737	0.707	0.000
kzwi	18	7	0.563	0.786	0.125
lang	20	6	0.667	0.729	0.036
mosl	27	3	0.692	0.667	0.000
mrbm	20	4	0.579	0.661	0.049
mrbn	21	4	0.875	0.688	0.000
mrbs	22	7	0.667	0.728	0.035
muet	20	4	0.800	0.695	0.000

oloo	20	3	0.667	0.675	0.005
opfi	21	6	0.529	0.663	0.081
piro	20	6	0.857	0.754	0.000
raue	7	4	0.400	0.733	0.192
rauw	53	6	0.739	0.718	0.000
schl	19	5	0.462	0.455	0.000
wolf	7	7	0.429	0.791	0.202
wtal	18	6	0.611	0.711	0.058
zurl	16	5	0.500	0.688	0.111
zurs	21	6	0.550	0.645	0.058
<u>average</u>	19.9		0.600	0.700	0.063

<u>Part 2</u>

	Population averages		
Population	A	Ho	He
allm	6.0	0.64	0.70
alls	8.6	0.62	0.70
amb1	8.4	0.46	0.68
amb3	9.3	0.56	0.68
amb4	7.1	0.52	0.68
birk	5.7	0.63	0.69
chap	7.7	0.49	0.60
ente	8.3	0.53	0.66
esch	8.6	0.55	0.67
flgu	8.1	0.64	0.68
fucu	8.4	0.44	0.66
grab	11.0	0.6	0.67
grut	4.7	0.53	0.68
gurw	6.9	0.34	0.61
haup	9.1	0.54	0.67
hell	8.3	0.55	0.67
hiwi	6.9	0.52	0.69
home	9.1	0.58	0.70
hubw	7.3	0.58	0.68
hund	8.1	0.6	0.67
insl	8.9	0.59	0.62
isol	7.3	0.63	0.68
jmai	8.6	0.58	0.72
jnw1	8.0	0.54	0.65
kzwi	8.1	0.52	0.71
lang	8.1	0.51	0.69
mosl	8.9	0.59	0.66
mrbm	6.4	0.53	0.64
mrbn	8.3	0.65	0.70
mrbs	8.7	0.61	0.66
muet	7.6	0.59	0.69
oloo	8.6	0.57	0.68
opfi	9.0	0.48	0.65
piro	7.0	0.59	0.67
raue	4.7	0.43	0.63

rauw	11.9	0.64	0.68
schl	8.0	0.51	0.62
wolf	5.6	0.55	0.69
wtal	8.0	0.53	0.68
zurl	7.6	0.53	0.67
zurs	8.6	0.54	0.66

Part 3

Locus	A	F_{ST}	F_{IS}
Ta1Ca1	13	0.022	0.012
Ta2Caga3	51	0.014	0.048
Ta3Caga2	9	0.034	-0.020
Ta4Caga4	20	0.010	0.148
Ta3Caga1	48	0.012	0.139
Ta6Ca1	9	0.015	0.436
Ta3Ca8	18	0.011	0.126
Average	24.0	0.0156	0.1299

Figure S1. Results of Beaumont & Nichols's (1996, *Proc. R. Soc. Lond. B* 263:1619-1626) test for selection for the eight *Rana temporaria* and seven *Triturus alpestris* microsatellite loci, under the stepwise mutation model. Predicted F_{ST} (solid line) and its 99% CI (dotted lines) were simulated using observed values for loci, population, individuals, and median F_{ST}. For *R. temporaria*, marker RtCa9 showed evidence of divergent selection and was therefore discarded from analyses. None of the *T. alpestris* markers showed any evidence of selection.

Linkage disequilibrium, not shown here, was rarely significant (Markov chain method described in Raymond & Rousset [1995, *Evolution* 49:1280-1283]; implemented in GENEPOP 4.0.10). There were 21 two-way tests for LD among loci within each population. For *R. temporaria*, none of these tests was significant at $\alpha = 0.05$ in 34 of the 48 populations, and all but one of the populations had significant results for ≤ 2 pairs of loci. For *T. alpestris*, an average of 1.0 of these tests was significant in each of the 41 populations, and all but three of the populations had significant results for ≤ 2 pairs of loci. This suggests that the markers were not closely linked.

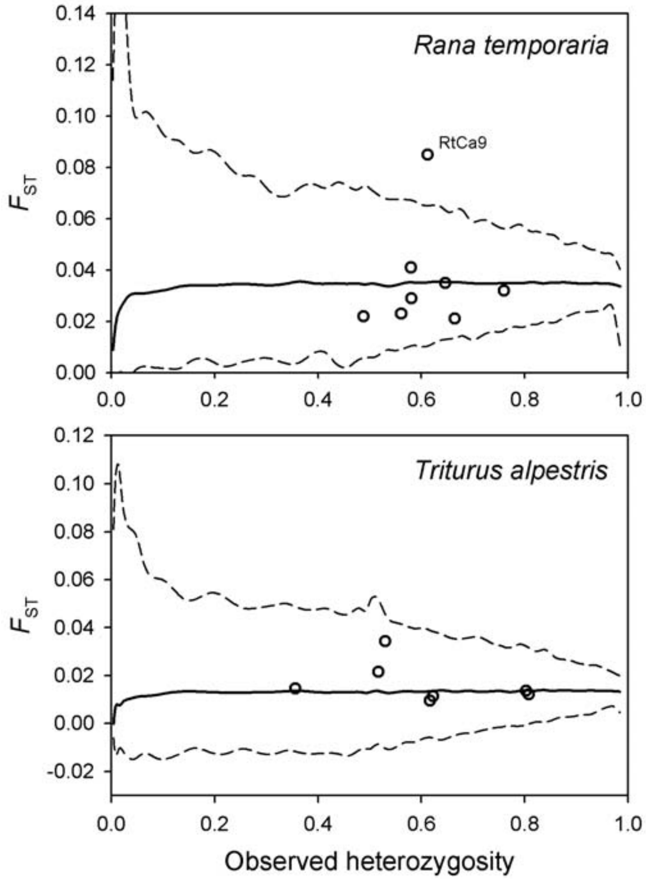

Figure S2. Isolation by distance among 48 populations of *Rana temporaria* (A) and 41 populations of *Triturus alpestris* (B) in northern Switzerland. Samples of both species were screened at the seven microsatellite loci described in Tables S2 and S3. The red line is a regression through the 1128 (*R. temporaria*) or 820 (*T. alpestris*) population pairs, which are indicated by the small black points. Blue points are averages (± 95% CI) of categories spaced approximately evenly on the logarithmic scale. In both species, isolation by distance was significant in Mantel tests comparing genetic divergence with the log of geographic distance (*R. temporaria*: $r = 0.236$, $P = 0.0002$; *T. alpestris*: $r = 0.143$, $P = 0.0021$; vegan package in R).

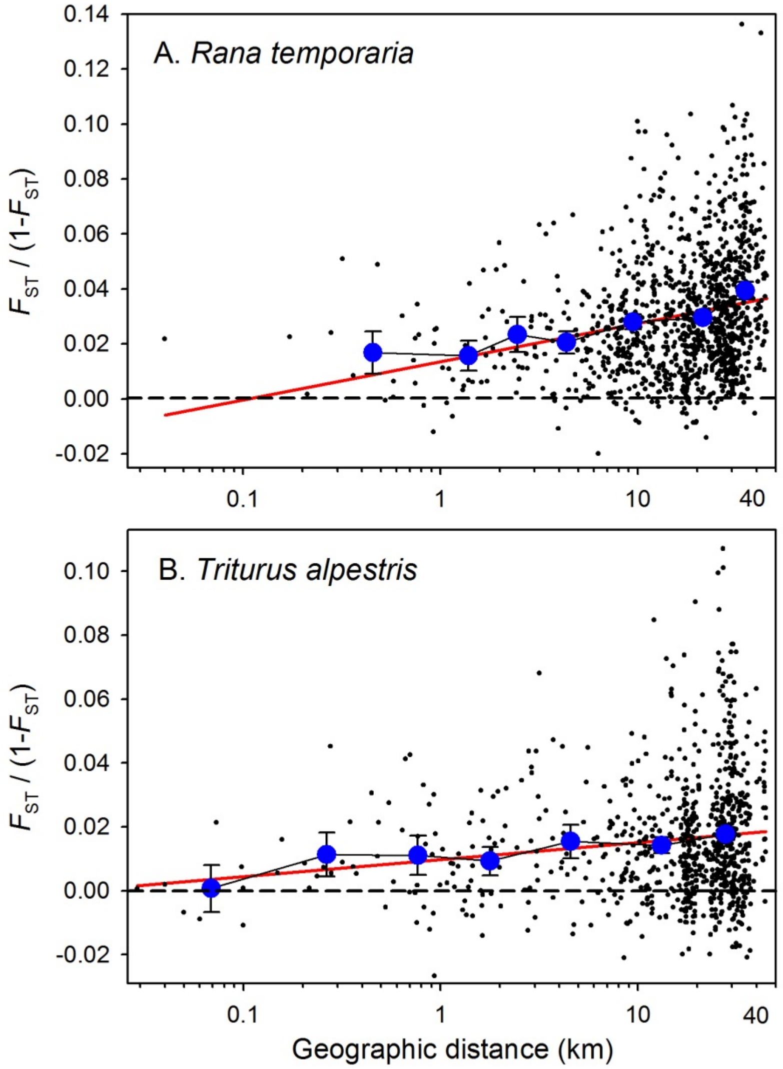

Supplement: Supplementary file 1 [file ece30002-3160-SD1.pdf]
