# Supplementary figures and images for: Permeability of the landscape matrix between amphibian breeding sites
Source: Ecol Evol. 2012 Nov 8;2(12):3160–7. doi: 10.1002/ece3.424 (PMC3539008; doi:10.1002/ece3.424)

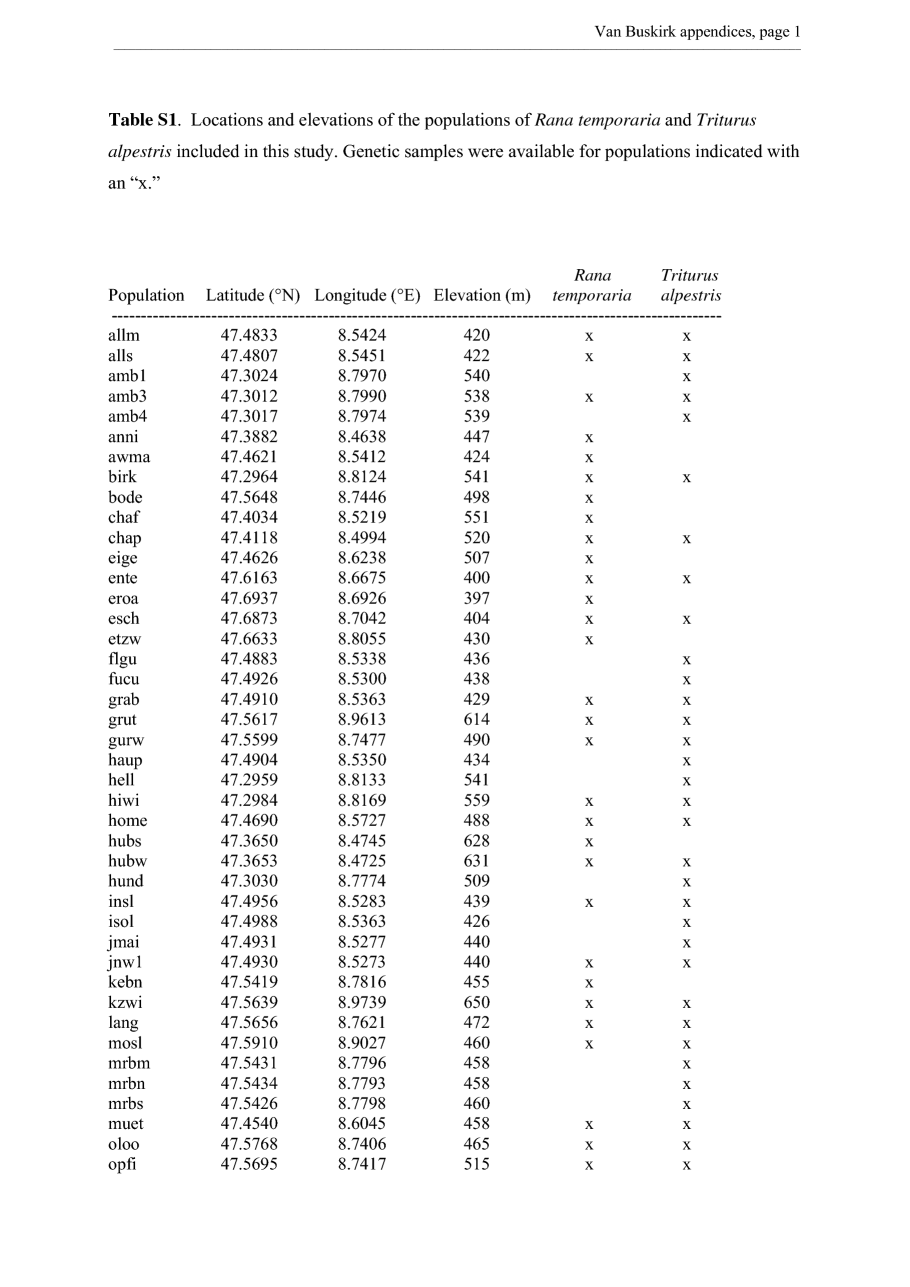

Supplement: Supplementary file 2 [file ece30002-3160-SD7.png]
